# Theoretical electronic structure with spin–orbit coupling effect of the molecules SrAt and BaAt for laser cooling studies

**DOI:** 10.1038/s41598-024-53564-5

**Published:** 2024-03-15

**Authors:** Amal Madi, Nayla El-Kork, Israa Zeid, Mahmoud Korek

**Affiliations:** 1 Faculty of Science, Beirut Arab University, P.O. Box 11-5020, Riad El Solh, Beirut, 1107 2809 Lebanon; 2 Department of Physics, Khalifa University, P.O. Box 127788, Abu Dhabi, United Arab Emirates; 3 Space and Planetary Science Center, Khalifa University, Abu Dhabi, United Arab Emirates

**Keywords:** Chemistry, Physics

## Abstract

Ab initio CASSCF/MRCI + Q calculations have been used to investigate the electronic structure and transition properties of the alkaline earth astatine molecules SrAt and BaAt. The adiabatic potential energy curves have been computed and plotted for the low-lying electronic states in the representations ^2S+1^Λ^+/−^ and Ω^(±)^ (with and without spin–orbit coupling effect). The spectroscopic and vibrational constants have been deduced for the corresponding bound states. An analysis of the Franck–Condon factors, the Einstein Coefficients, and the branching ratios among different vibrational levels has shown that both SrAt and BaAt molecules are suitable candidates for Doppler and Sysphus laser cooling. Experimental laser cooling schemes and conditions for these two molecules have been proposed. These results may pave the way for new spectroscopic and laser cooling experiments of alkaline earth astatine molecules.

## Introduction

Researchers have been interested in the spectroscopic studies of the alkali and alkaline earth halides^1,2^ because of their relevance to astrophysics. These have been detected in the interstellar medium^3^ and the upper atmosphere^4^. In this view, MgF, SrF^1^, and MgCl molecules are predicted to appear in S-stars^2^, on the sun’s surface, and in the sunspot’s spectrum. Moreover, these alkaline-earth mono-halide molecules are highly interesting for high-temperature reactions in catalysis and corrosion processes^5^.

From the perspective of laser cooling experiments, the compounds of alkaline-earth metals have been proposed as promising candidates for laser cooling and controlling the preparation of many-body entangled states^6–8^. SrF and YO^9,10^ molecules have been cooled using transverse cooling methods, while CaF has been cooled by longitudinal laser cooling^11^. Extensive theoretical studies have also been performed for molecules that possess similar electronic structures, such as BeF^12^ and MgF^13^

The electronic structure of the alkaline-earth halide molecules, including MgAt, has been studied in the literature. The first few low-lying excited electronic states of the molecules MgCl, MgBr, and MgI have already been investigated^14–19^. In 2015, Wan et al*.*^20^ presented for BeI and MgI an ab initio investigation for the effect of spin–orbit coupling on laser cooling, where they calculated the spectroscopic properties and the cooling wavelength of these molecules in the ultra-violet region. The suitability of laser cooling of alkaline earth mono halides BaX (X = F, Cl, Br, I) and MgX(X = Br, At, I) has been verified respectively by Yang et al.^21^ and Yang and Tao^22^.

We present a theoretical study by using the ab initio method (CASSCF/MRCI + Q) for the molecules SrAt and BaAt to test the candidacy of alkaline-earth astatine species for laser cooling. Section “Computational approach” includes the computational approach followed for the pursued computations. The adiabatic potential energy curves, the dipole moment curves of the low-lying doublet and quartet electronic states, and their spectroscopic constants in the ^2S+1^Λ^+/−^ and Ω^(±)^ representations are presented in Section “Potential energy curves, spectroscopic parameters, and permanent dipole moment curves”. In addition, the vibrational energy E_v_, the ro-vibrational constants B_v_, D_v,_ the abscissa of the turning points R_min,_ and R_max_ of the ground, and the bound excited electronic states are displayed in Section “The ro-vibrational parameters”. Section “Laser cooling study of SrAt and BaAt molecules” includes a laser cooling investigation of the molecules SrAt and BaAt, done by calculating the Franck–Condon Factors (FCF), the Einstein Coefficients, the radiative lifetime, and the branching ratio among specific vibrational levels. Experimental parameters are presented, including the minimum slowing distance, the Doppler and recoil temperatures, and the maximal deceleration of the molecules. Laser cooling schemes for the molecules SrAt and BaAt are presented with three and four lasers in the visible and near-infrared regions, respectively.

## Computational approach

The Complete Active Space Self Consistent Field (CASSCF) has been used as a reference for generating the multiconfiguration wavefunctions of the considered two molecules. It is followed by the Multireference configuration interaction (MRCI) method, with Davidson correction (+ Q)^23^. The current calculations are done by employing the MOLPRO program package^24^, taking advantage of the graphical user interface GABEDIT^25^ to study the electronic structure of the electronic states of SrAt and BaAt in the doublet and quartet multiplicities with and without considering the spin–orbit coupling effect. For the BaAt molecule, the electronic wavefunctions of seventy-eight core electrons of At are described by the quasi-relativistic effective core potential ECP78MWB^26^ for *s, p, d* functions, while for the SrAt, they are described by ECP60MDF. Thirty-six electrons of Sr were frozen using the ECP36SDF for *s, p* functions, and 46 electrons of Ba were frozen using the ECP46MWB for *s, p, d* functions. It is worth noting that the C_ꝏv_ group was decomposed into C_2v_ sub-group because of the limitations of the MOLPRO software. Table 1 reports the active space orbitals for the two considered molecules. Thus, the molecular orbitals are labeled in the irreducible representation as 4*a1*, 1*b1*, 1*b2*, and 0*a2* for SrAt denoted by [4,1,1,0], and 6*a1*, 3*b1*, 3*b2*, and 1*a2* denoted by^1,3,3,6^ for BaAt. Also, the molecules SrAt and BaAt have been investigated in spin–orbit Ω^(±)^ representation where Sr is treated as a system of 10 electrons using ECP28MDF^27^, Ba is treated by ECP46MDF ^28^, and At is treated by ECP60MDF for SrAt molecule and ECP78MDF for BaAt molecule^28^. Then the active space in the spin–orbit calculations of SrAt becomes *4σ (Sr: 5s; At:6p*_*0*_*, 6s, 7s), 1π (Sr:0; At: 6p*_*1*±_*), 0δ* and that of BaAt is *6σ (Ba: 5d*_*0*_*, 5d*_+*2,*_*6p*_*0,*_* 6 s; At:7 s, 6p*_*0*_*), 3π (Ba:5d*_±*1*_*, 5p*_±*1*_*; At: 6p*_±*1*_*),1δ (Ba: 5d*_*-2*_*)* and the molecular orbitals are labeled as [4,1,1,0] for SrAt and [1,3,36] for BaAt.

**Table 1:** The active space orbitals for the SrAt and BaAt molecules.

Molecule	Orbitals of active space
SrAt	*4σ (Sr: 5s; At:6p*_*0*_*, 6s, 7s), 1π (Sr:0; At: 6p*_*1*±_*), 0δ*
BaAt	*6σ (Ba: 5d*_*0*_*, 5d*_+*2,*_*6p*_*0,*_* 6 s; At:7 s, 6p*_*0*_*), 3π (Ba:5d*_±*1*_*, 5p*_±*1*_*; At: 6p*_±*1*_*),1δ (Ba: 5d*_*-2*_*)*

Additionally, the potential energy curves of the molecules BeAt, MgAt, and CaAt have been computed for a spectroscopic trend comparison (see Section “Potential energy curves, spectroscopic parameters, and permanent dipole moment curves”). The basis set is cc-pV5Z^29^ for Be and Mg atoms and ECP10MWM^30^ for Ca atom. For At atom, the same basis set (ECP78MDF) was used among all molecules. The corresponding potential energy curves of the doublet and quartet electronic states for the considered five molecules are given in Figs. FS1-FS9 along with their static dipole moments (Figs. FS10–FS15) in the supplementary materials.

## Results and discussion

### Potential energy curves, spectroscopic parameters, and permanent dipole moment curves

**Figure 1:**
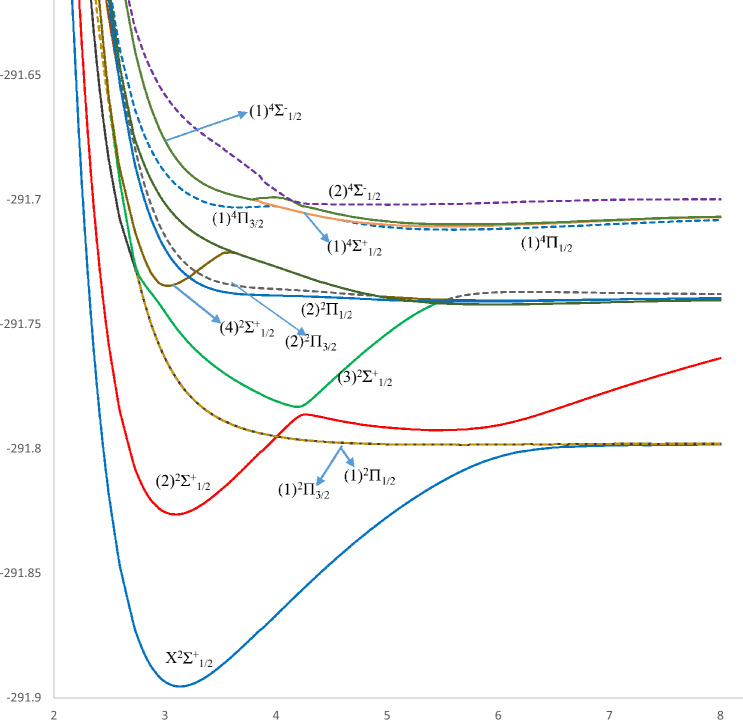
Potential energy curves of the lowest Ω^(±)^ doublet and quartet states of the SrAt molecule.

**Figure 2:**
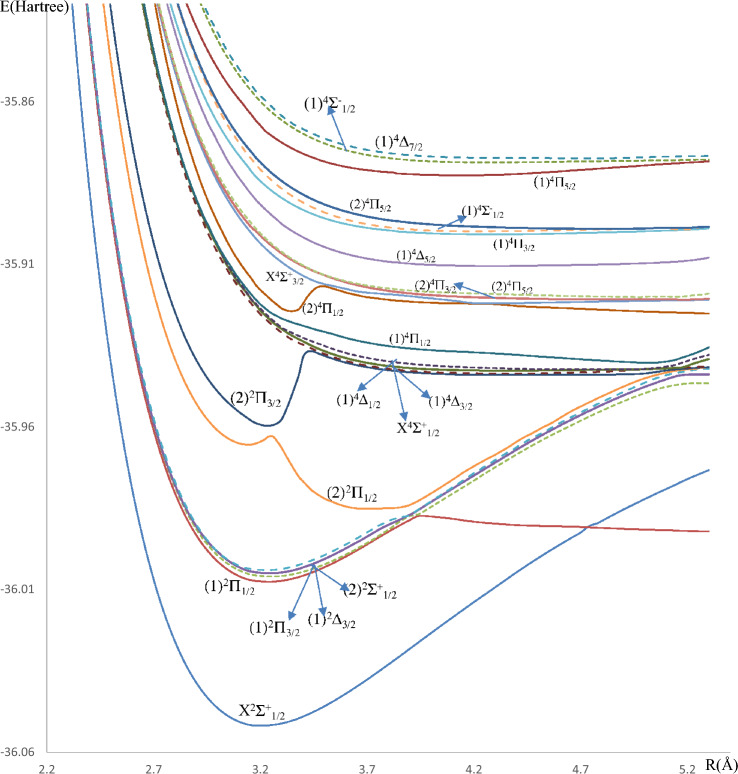
Potential energy curves of the lowest Ω^(±)^ doublet and quartet states of the BaAt molecule.

The ab initio method employed in the present work allowed the investigation of the adiabatic potential energy curves (PECs) of the electronic states of the alkaline earth astatine molecules SrAt and BaAt in their doublet and quartet multiplicities. The PECs of thirty-five electronic states (eight doublet and five quartet states of SrAt molecule) and (seven doublet and 15 quartet states of BaAt molecule), taking into account spin–orbit coupling, in the representation Ω^(±)^ are provided in Figs. 1, 2 as a function of the internuclear distance R, while the PECs of 28 states (eight doublet and ten quartet states of SrAt) and (five doublet and five quartet states of BaAT) calculated without considering this effect are given in the supplementary material in Figs. FS5–FS8. One can notice that the two molecules have deep potential wells reflecting a dominancy of the attractive forces within the molecule’s constituents and shallower ones reflecting the dominancy of repulsive forces. Additionally, many unbound repulsive states are observed. The ground state is X^2^∑^+^, which has a deep potential well for the two molecules. The spectroscopic parameters T_e_, R_e_, ${\upomega }_{{\text{e}}}$, and B_e_ have been calculated for the bound states upon fitting their potential energy curves into a polynomial around the equilibrium position R_e_. The calculated spectroscopic parameters of BaAt and SrAt molecules with and without spin–orbit coupling effects are listed in Tables 2 and 3. The data we present here has been calculated for the first time, so comparing it with the literature is not possible. Still, the validity of the spectroscopic constants can be confirmed in Table 4 through the homogeneous trend of T_e_, R_e,_ and ω_e_ of the ground and some of the low-lying electronic states of the molecules BeAt, MgAt, CaAt, SrAt, and BaAt, as in previously published work^31^. The correct trend of the spectroscopic constants is evident for all the investigated electronic states: an increase in the atomic mass of the alkaline earth atom corresponds to a decrease in the electronegativity, which leads to an increase in the equilibrium bond length R_e_, a decrease in the transition energy T_e_, and the harmonic frequency ω_e_. The spectroscopic constants are not calculated for the remainder of the excited states because they are either unbound states, have very shallow potential wells, or present an avoided crossing behavior near their minimum.

**Table 2:** Spectroscopic constants of the molecules SrAt and BaAt without spin–orbit coupling calculated by using the multireference configuration interaction technique.

State	T_e_ (cm^−1^)	R_e_ (Å)	ω_e_ (cm^−1^)	B_e_ (cm^−1^)
X^2^∑^+^	0.00	3.136	145.9	00.277
(2)^2^∑^+^	15,147.24	3.096	147.3	0.0284
(3)^2^∑^+^	24,615.22	4.185	196.5	0.0156
(4)^2^∑^+^	35,286.13	3.024	195.9	0.0298

State	T_e_ (cm^**−**1^)	R_e_ (Å)	ω_e_ (cm^−1^)	B_e_ × 10 (cm^−1^)
X^2^Σ^+^	0.00	3.202	117.5	0.1980
(2)^2^Σ^+^	9997.33	3.242	118.2	0.1930
(1)^2^Δ	10,090.93	3.263	107.1	0.1905
(1)^2^Π	10,297.57	3.223	104.7	0.1956

**Table 3:** Spectroscopic constants of the molecules SrAt and BaAt with spin–orbit coupling effects taken into consideration, calculated by using the multireference configuration interaction technique.

SrAt with spin–orbit coupling					
State	T_e_ (cm^−1^)	R_e_ (Å)	ω_e_ (cm^−1^)	B_e_ × 10 (cm^−1^)	
X^2^Σ^+^_1/2_	0.00^b^	3.136	145.7	0.2770	
(2)^2^$\sum_{1/2}^{ + }$	15,146.43^b^	3.097	148.3	0.0284	
(3)^2^$\sum_{1/2}^{ + }$	24,614.95^b^	4.185	196.5	0.0156	
(3)^2^$\sum_{1/2}^{ + }$	35,283.30	3.024	200.5	0.0298	

BaAt with spin–orbit coupling					
State	T_e_ (cm^**−1**^)	R_e_ (Å)	ω_e_ (cm^**−1**^)	B_e_ × 10 (cm^**−1**^)	µ_e_ (a.u)
X^2^Σ^+^_1/2_	0.00	3.203	121.2	0.1978	2.068
(1)^2^Π_1/2_	9692.61	3.239	108.4	0.1935	2.384
(1)^2^Π_3/2_	10,045.89	3.256	108.9	0.1915	3.660
(1)^2^Δ_5/2_	10,266.99	3.244	104.1	0.1928	3.005
(2)^2^$\sum_{ - 1/2}^{ + }$	10,506.81	3.220	103.1	0.1959	2.567
(2)^2^Π_3/2_	14,612	3.725	295.9	0.1464	0.770

**Table 4:** Spectroscopic constants trends among different electronic states of the molecules BeAt, MgAt, CaAt, SrAt, BaAt.

States	constants	BeAt	MgAt	CaAt	SrAt	BaAt	Trend
X^2^∑^+^	T_e_ (cm^−1^)	0.0	0.0	0.0	0.0	0.0	↘
	R_e_(Å)	2.269	2.684	3.002	3.136	3.294	↗
	ω_e_ (cm^-1^)	658.2	290.6	210.7	145.9	117.5	↘
(1)^2^Π	T_e_ (cm^−1^)					10,297.57	↘
	R_e_(Å)					3.265	↗
	ω_e_ (cm^-1^)					104.7	↘
(2)^2^Π	T_e_ (cm^−1^)	28,160.0	24,640.5				↘
	R_e_(Å)	2.406	2.754				↗
	ω_e_ (cm^-1^)	909.7	450.5				↘
(1)^4^∑^+^	T_e_ (cm^−1^)	33,471.5	34,027.5				↘
	R_e_(Å)	2.792	3.199				↗
	ω_e_ (cm^-1^)	155.9	124.8				↘
(1)^4^Δ	T_e_ (cm^−1^)	34,368.7					↘
	R_e_(Å)	3.010					↗
	ω_e_ (cm^-1^)	99.8					↘
	B_e_(cm^-1^)	0.02137					↘
(2)^2^∑^+^	T_e_ (cm^−1^)	37,363.2		32,681.9	15,147.24	9997.33	↘
	R_e_(Å)	3.179		3.181	3.096	3.242	↗
	ω_e_ (cm^-1^)	167.2		141.4	147.3	118.2	
(2)^4^∑^+^	T_e_ (cm^−1^)	63,998.3	34,867.0				↘
	R_e_(Å)	2.404	3.306				↗
	ω_e_ (cm^-1^)	479.2	97.7				↘

Moreover, By using the basis set ECP60MDF For At atom, the comparison of our spectroscopic constants for MgAt (Table 4) with those given by Yang and Gao^22^ shows a very-good agreement with relative differences of 0.6%, 1.8%, and 2.5%, respectively, for ΔR_e_/R_e_, Δω_e_/ω_e_, ΔB_e_/B_e_ for the ground state X^2^∑^+^. For (2)^2^Π states, these relative differences are 1.63%, 1.60%, 3.70%, and 3.6% for ΔT_e_/T_e_, ΔR_e_/R_e_, Δω_e_/ω_e_, and ΔB_e_/B_e,_ respectively.

Given the correct trend and the very good agreement of our spectroscopic constants with those available in the literature^22^, we may confirm the accuracy of our results for the two molecules, SrAt and BaAt.

The permanent dipole moment curves (PDMCs) are an effective tool for understanding the polarity and the strength of the long-range dipole–dipole forces in diatomic molecules. The permanent dipole moment curves (PDMCs) of the five molecules, BeAt, MgAt, CaAt, SrAt, and BaAt (without including the spin–orbit coupling effects), are represented in Figs. FS10–FS15 of the supplementary material. The electrons’ density distribution can be understood according to the polarity of the dipole moments ranging from − µ to + µ. The dipole moment usually exhibits positive values when the electrons’ density is closer to the alkaline earth metal considered at the origin. On the contrary, flipping in the polarity occurs when the dipole moment becomes negative as the electrons’ density becomes closer to the At atom. Consequently, the positive values of dipole moments can be denoted by Sr^δ−^At^δ+^ and Ba^δ−^At^δ+^. The values of dipole moment, which tend to be zero at large internuclear distances, are evidence of the molecule’s dissociation into neutral fragments. In contrast, those with constant values indicate dissociation into ionic fragments.

### The ro-vibrational parameters

The theoretical determination of a given level’s rovibrational constants is effective in the prediction process of absorption/emission line positions. These are useful in guiding experimental investigations that facilitate the detection of unknown molecules. In the conventional approach of the Rayleigh-Schrödinger perturbation theory (RSPT), the first analytical expressions of the centrifugal distortion constants (CDC) have been derived by Albritton et al.^32^. To overcome the complexity of the computation of such expressions, Hutson derived an algorithm^33^ by using the Numerov difference equation for the determination of the constants ${D}_{\upnu }$, ${H}_{\upnu }$, ${L}_{\upnu }$, and ${M}_{\upnu }$ in terms of the vibrational wave function ${\Psi }_{\upnu }$. But in this algorithm, some difficulties had appeared for some potentials (like the Lennard–Jones potential), such as the problem of treating high vibrational levels near the dissociation limit. An improvement has then been introduced to the Huston algorithm Tellinghuisen^34^, but it is still insufficient to reach larger orders of centrifugal distortion constants. For this purpose, the quantum mechanical canonical function method^35–37^ was developed to calculate the rotation–vibration constants for highly excited electronic states with many centrifugal distortion constants.

This approach is used in the present work to determine the rovibrational parameters of the BaAt molecules, including the vibrational energy E_v_, the rotational constant B_v_, the centrifugal distortion constant D_v_, and the abscissas of the turning point R_min_ and R_max._ These values, including the spin–orbit coupling effects, are given in (Tables 5, 6). Since most states are unbound, the spectroscopic constants and the ro-vibrational parameter of the quartet spin–orbit potential energy curves have not been calculated. There are no comparisons with other results because these constants are calculated here for the first time.

**Table 5:** The rovibrational constants for the different vibrational levels of the ground state X^2^$\sum_{1/2}^{ + }$ of BaAt molecule calculated with the spin–orbit coupling effects taken into account.

X^2^$\sum_{1/2}^{ + }$ (BaAt)					
v	E_v_	B_v_ × 10^2^	D_v_ × 10^–9^	R_min_	R_max_
0	64.82	1.9851	2.1390	3.1445	3.2665
1	185.23	1.9757	1.4684	3.1150	3.3109
2	315.43	1.9666	1.7921	3.0918	3.3439
3	445.32	1.9648	1.9265	3.0730	3.3711
4	573.87	1.9604	1.5331	3.0569	3.3936
5	704.15	1.9552	1.8944	3.0425	3.4150
6	833.34	1.9531	1.8371	3.0296	3.4351
7	961.84	1.9452	1.7062	3.0178	3.4539
8	1090.29	1.9430	2.0408	3.0067	3.4718
9	1217.45	1.9389	1.4308	2.9968	3.4888
10	1345.50	1.9325	2.1397	2.9872	3.5052
11	1472.00	1.9313	1.6957	2.9782	3.5210
12	1598.51	1.9245	1.5957	2.9697	3.5363
13	1725.12	1.9214	2.2275	2.9616	3.5513
14	1850.29	1.9178	1.4225	2.9539	3.5658
15	1975.94	1.9116	1.9724	2.9465	3.5801
16	2100.82	1.9096	1.9296	2.9395	3.5940
17	2225.09	1.9041	1.5239	2.9327	3.6077
18	2349.46	1.8997	2.0964	2.9261	3.6211
19	2472.95	1.8969	1.7142	2.9198	3.6342
20	2596.21	1.8909	1.7114	2.9137	3.6473
21	2719.20	1.8877	2.1024	2.9078	3.6602
22	2841.37	1.8835	1.6343	2.9021	3.6729
23	2963.38	1.8781	1.8345	2.8966	3.6853
24	3084.98	1.8754	1.9467	2.8912	3.6976
25	3206.08	1.8711	1.5591	2.8860	3.7098
26	3327.10	1.8663	1.9931	2.8809	3.7219
27	3447.51	1.8629	1.9307	2.8759	3.7338
28	3567.40	1.8581	1.5868	2.8711	3.7456
29	3687.16	1.8540	1.9683	2.8664	3.7573
30	3806.40	1.8506	1.8759	2.8618	3.7689
31	3925.19	1.8456	1.6647	2.8573	3.7805
32	4043.74	1.8416	1.9715	2.8529	3.7919
33	4161.78	1.8382	1.8197	2.8486	3.8033
34	4279.45	1.8332	1.7000	2.8444	3.8145
35	4396.83	1.8293	1.9745	2.8403	3.8257
36	4513.72	1.8257	1.7975	2.8363	3.8368
37	4630.26	1.8209	1.7018	2.8324	3.8479
38	4746.50	1.8169	1.9625	2.8286	3.8589
39	4862.28	1.8133	1.7952	2.8248	3.8698
40	4977.70	1.8087	1.6965	2.8211	3.8807
41	5092.84	1.8046	1.9647	2.8175	3.8915
42	5207.51	1.8009	1.8381	2.8139	3.9023
43	5321.80	1.7964	1.7153	2.8104	3.9130
44	5435.78	1.7923	1.9637	2.8069	3.9237
45	5549.32	1.7887	1.8835	2.8035	3.9343
46	5662.45	1.7843	1.7285	2.8002	3.9449
47	5775.26	1.7800	1.9460	2.7969	3.9555
48	5887.64	1.7763	1.9334	2.7937	3.9661
49	5999.59	1.7720	1.7369	2.7905	3.9766
50	6111.22	1.7676	1.8938	2.7874	3.9871
51	6222.43	1.7638	1.9673	2.7843	3.9976
52	6333.21	1.7596	1.7644	2.7813	4.0080
53	6443.64	1.7551	1.8367	2.7783	4.0184
54	6553.70	1.7512	1.9903	2.7754	4.0289
55	6663.30	1.7472	1.8281	2.7725	4.0393
56	6772.54	1.7427	1.7932	2.7667	4.0496
57	6881.42	1.7386	1.9795	2.7669	4.0600
58	6989.87	1.7347	1.9102	2.7641	4.0703
59	7097.92	1.7304	1.7744	2.7614	4.0807
60	7205.63	1.7261	1.9188	2.7587	4.0910
61	7312.93	1.7222	1.9728	2.7560	4.1013
62	7419.82	1.7181	1.8031	2.7534	4.1115
63	7526.35	1.7137	1.8333	2.7509	4.1218
64	7632.52	1.7097	1.9834	2.7483	4.1320
65	7738.28	1.7058	1.8830	2.7458	4.1423
66	7843.66	1.7015	1.7825	2.7433	4.1525
67	7948.70	1.6973	1.9229	2.7409	4.1627
68	8053.35	2.7646	16.474	2.7385	4.1729
69	8157.61	2.7510	19.072	2.7361	4.1830
70	8261.52	2.7248	38.951	2.7337	4.1932
71	8365.08	2.8256	0.7727	2.7314	4.2033

**Table 6:** The rovibrational constants of different vibrational levels of some excited states of BaAt molecule calculated with the spin–orbit coupling effect taken into account.

(1)^2^Π_1/2_(BaAt)					
v	E_v_	B_v_ × 10^2^	D_v_ × 10^9^	R_min_	R_max_
0	58.24	1.9478	2.1836	3.1769	3.2971
1	174.54	1.9469	1.9553	3.1360	3.3413
2	293.63	1.9391	1.7052	3.1136	3.3737
3	415.70	1.9324	1.8347	3.0944	3.4009
4	538.16	1.9276	1.9325	3.0780	3.4256
5	660.13	1.9232	2.0188	3.0635	3.4480
6	781.21	1.9187	1.8620	3.0505	3.4686
7	902.20	1.9133	1.8055	3.0385	3.4881
8	1143.36	1.9048	1.9177	3.0172	3.5245
9	1263.12	1.8996	1.8493	3.0076	3.5416
10	1382.67	1.8949	1.9840	2.9985	3.5581
11	1501.66	1.8908	1.9334	2.9899	3.5742
12	1620.24	1.8860	1.9422	2.9818	3.5898
13	1738.37	1.8814	1.8951	2.9740	3.6050
14	1856.17	1.8769	1.9719	2.9665	3.6200
15	1973.48	1.8724	1.9575	2.9594	3.6345
16	2090.35	1.8679	1.9008	2.9526	3.6489
17	2206.86	1.8632	1.9743	2.9460	3.6630
18	2322.89	1.8588	1.9654	2.9396	3.6769
19	2438.49	1.8542	1.9079	2.9335	3.6905
20	2553.72	1.8497	1.9523	2.9276	3.7040
21	2668.54	1.8454	1.9569	2.9218	3.7173
22	278,294	1.8408	1.9611	2.9163	3.7305
23	2896.89	1.8361	2.0101	2.9109	3.7433
24	3010.34	1.8313	2.0413	2.9056	3.7561
25	3123.23	1.8259	2.1577	2.9005	3.7697
26	3235.38	1.8200	2.2979	2.8956	3.7833
27	3346.62	1.8137	2.2723	2.8908	3.7959
28	3457.00	1.8076	2.1981	2.8861	3.8092
29	3566.67	1.8021	2.2145	2.8816	3.8232
30	3675.61	1.7960	2.4310	2.8772	3.8370
31	3783.56	1.7884	2.5940	2.8729	3.8503
32	3890.37	1.7816	2.2303	2.8688	3.8635
33	3996.53	1.7769	2.0678	2.8647	3.8774
34	4102.19	1.7703	2.8685	2.8608	3.8920

(1)^2^Π_3/2_ (BaAt)					
v	E_v_	B_v_ × 10^2^	D_v_ × 10^9^	R_min_	R_max_
0	55.68	1.9325	2.3756	3.1891	3.3116
1	165.91	1.9327	2.1183	3.1455	3.3565
2	278.92	1.9246	1.7657	3.1227	3.3893
3	395.49	1.9168	1.8988	3.1036	3.4174
4	512.82	1.9116	2.0688	3.0871	3.4425
5	629.60	1.9074	2.0895	3.0726	3.4654
6	745.79	1.9023	1.9515	3.0594	3.4869
7	861.91	1.8968	1.9885	3.0474	3.5066
8	1093.49	1.8884	1.9580	3.0260	3.5436
9	1208.97	1.8834	2.0799	3.0163	3.5610
10	1323.87	1.8789	1.9376	3.0071	3.5777
11	1438.60	1.8742	1.9660	2.9985	3.5941
12	1667.04	1.8655	1.8567	2.9824	3.6256
13	1780.98	1.8615	1.8832	2.9749	3.6405
14	1894.84	1.8583	1.8103	2.9677	3.6535
15	2008.75	1.8552	1.7406	2.9608	3.6671
16	2122.78	1.8513	2.0840	2.9541	3.6819
17	2236.22	1.8456	2.2751	2.9476	3.6961
18	2348.69	1.8388	2.1811	2.9414	3.7102
19	2460.43	1.8350	1.6162	2.9355	3.7235
20	2572.51	1.8343	1.3160	2.9297	3.7355
21	2685.34	1.8322	1.7914	2.9240	3.7472
22	2798.12	1.8283	1.9598	2.9184	3.7592
23	2910.55	1.8246	1.7537	2.9131	3.7720
24	3022.89	1.8204	2.1463	2.9078	3.7846
25	3134.52	1.8144	2.2322	2.9028	3.7966
26	3245.45	1.8106	1.3770	2.8978	3.8084
27	3356.80	1.8103	1.2933	2.8930	3.8196
28	3468.72	1.8083	1.7837	2.8882	3.8297
29	3580.50	1.8044	1.7852	2.8836	3.8400
30	3692.10	1.8009	1.9773	2.8791	3.8518
31	3803.22	1.7950	2.6085	2.8746	3.8640
32	3913.06	1.7852	3.3918	2.8703	3.8756
33	4020.62	1.7707	4.7762	2.8662	3.8886
34	4124.32	1.7478	6.9259	2.8623	3.9127
35	4221.99	1.7155	7.9905	2.8586	3.9444

### Laser cooling study of SrAt and BaAt molecules

The difference in equilibrium positions ΔR_e_ between the ground state X^2^∑^+^ and the two excites states (1)^2^Π and (2)^2^∑^+^ states of SrAt and BaAt are minimal; this directed our attention to verify the laser cooling suitability for these molecules through cycles involving the aforementioned states, in the Ω^(±)^ representation. However, an experimental confirmation of the presented electronic structure calculation is highly recommended before such step is taken.

**Figure 3:**
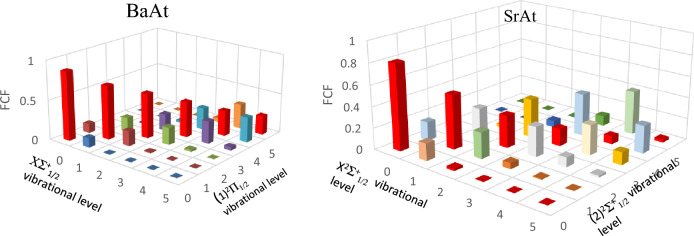
Franck–Condon factor for the transitions X^2^$\sum_{1/2}^{ + }$ − (2)^2^∑_1/2_ and X^2^$\sum_{1/2}^{ + }$ − (1)^2^Π_1/2_ of the molecules SrAt and BaAt, respectively.

The main criterion for keeping a molecule in a closed-loop cycle is a highly diagonal Franck–Condon factor (FCF) among the lowest vibrational levels of a bound excited state and those of the ground state ^38^. The vibrational FCF of the transition X^2^∑^+^_1/2_—(1)^2^$\sum_{1/2}^{ + }$ of the molecule SrAt (calculated by using the LEVEL 11 program^39^) is plotted in Fig. 3. One can notice that the transition among the vibrational levels v′ = v = 0 has a higher probability than the remaining ones. At the same time, the deexcitation of the vibrational level v′ = 0 takes place mainly through the channel v′_0_v_1,_ v′_0_v_2_, and v′_0_v_3_ with the following FCF, respectively f_0′0_ = 0.812067, f_0′1_ = 0.161978, f_0′2_ = 0.022776 and f_0′3_ = 0.002822. The deexcitation through the remaining channels is minimal.

**Figure 4:**
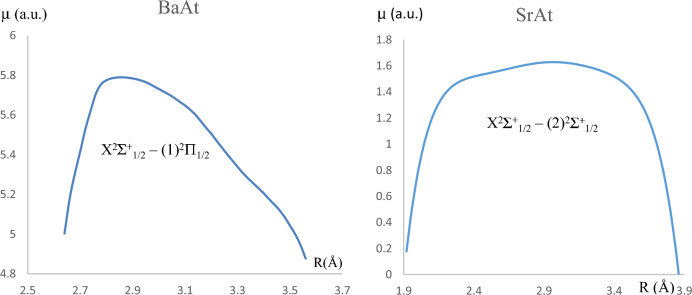
Transition dipole moments for the transitions X^2^$\sum_{1/2}^{ + }$ − (2)^2^$\sum_{1/2}^{ + }$ and X^2^$\sum_{1/2}^{ + }$ − (1)^2^Π_1/2_ of the molecules SrAt and BaAt, respectively.

A short radiative lifetime among vibrational levels involved in the cooling cycle is the second criterion for a successful laser cooling process, as it maximizes the cooling rate and produces a strong Doppler force. This can be done by calculating the vibrational Einstein coefficient A_ν′ν_ given by^40^

1 $$A_{{v^{\prime}v}} = \frac{{\left( {3.1361891} \right)\left( {10^{ - 7} } \right)(\Delta E)^{3} \left( {\left\langle {\left. {\psi_{{\nu^{\prime}}} } \right|M\left( r \right)\left| {\psi_{\nu } } \right.} \right\rangle } \right)^{2} }}{2}$$

where M(r) is the electronic transition dipole moment (in Debye), and ΔE is the energy difference between the two studied electronic states. The computed X^2^$\sum_{1/2}^{ + }$ − (1)^2^$\sum_{1/2}^{ + }$ transition dipole moment is represented in Fig. 4. The radiative lifetimes (given by $\tau_{{v^{\prime}}} = \frac{1}{{\mathop \sum \nolimits_{v} A_{{v^{\prime}v}} }}$) of six considered vibrational levels (*v*′), and the vibrational branching ratio (given by R_v′v_ = $\frac{{A_{{v^{\prime}v}} }}{{\mathop \sum \nolimits_{v} A_{{v^{\prime}v}} }}$^41,42^) among the vibrational transitions between different levels (*v*′) and (*v*) are displayed in Table 7. The transition X^2^$\sum_{1/2}^{ + }$ − (1)^2^$\sum_{1/2}^{ + }$ of SrAt molecule satisfies this condition, given the short radiative lifetimes that vary as 92.50 ns ≤ τ ≤ 101.9 ns among different values of *v*′*.*

**Table 7:** The radiative lifetimes τ, and the vibrational branching ratio of the vibrational transitions between the electronic states (2)^2^$\sum_{1/2}^{ + }$ − X^2^$\sum_{1/2}^{ + }$ of the molecule SrAt.

SrAt X^2^$\sum_{1/2}^{ + }$—(2)^2^$\sum_{1/2}^{ + }$								
	ν′((2)^2^$\sum_{1/2}^{ + }$) = 0	1	2	3	4	5	6	
v (X^2^$\sum_{1/2}^{ + }$) = 0	A_νν′_	7,442,918.232	3,273,038	230,102.8869	5542.5452	31.718372	1.6255315	0.0611421
	R_νν′_	0.68846092	0.17950205	0.01266762	0.00030798	0.00000186	0.00000012	0.00000001
ν = 1	A_νν′_	2,914,995.722	9,348,556.9	5,417,934.756	624,865.87	21,793.435	168.92822	0.6511433
	R_νν′_	0.26963357	0.51269957	0.29826807	0.03472158	0.00127641	0.00001222	0.00000007
ν = 2	A_νν′_	398,442.1537	4,465,885	5,505,289.272	6,717,603.4	1,135,179.9	52,382.551	567.34583
	R_νν′_	0.03685542	0.24492094	0.30307711	0.37327342	0.06648574	0.00378933	0.00005781
ν = 3	A_νν′_	48,541.45396	965,817.19	5,076,622.637	2,927,379.1	7,369,917	1,711,139.5	97,718.903
	R_νν′_	0.00449003	0.05296797	0.27947816	0.16266408	0.43164467	0.12378322	0.00995751
ν = 4	A_νν′_	5511.056373	156,636.02	1,558,285.775	5,098,609.6	1,338,072.3	7,526,705	2,280,078.3
	R_νν′_	0.00050977	0.00859033	0.08578673	0.28331167	0.07836883	0.54447913	0.23233895
ν = 5	A_νν′_	516.0675057	21,443.115	321,989.6795	2,089,996.4	4,698,100.3	465,627.24	7,363,548
	R_νν′_	0.00004774	0.00117600	0.01772617	0.11613369	0.27516049	0.03368331	0.75034222
ν = 6	A_νν′_	27.76708477	2610.0316	54,424.11173	532,471.69	2,510,942	4,067,654.5	71,672.885
	R_νν′_	0.00000257	0.00014314	0.00299616	0.02958757	0.14706200	0.29425267	0.00730343
Sum(s^−1^) = A_ν′ν_		10,810,952.45	18,233,986	18,164,649.12	17,996,469	17,074,037	13,823,679	9,813,586.1
τ (s) = 1/A_ν′ν_		9.24988E−08	5.484E−08	5.5052E−08	5.557E−08	5.857E−08	7.234E−08	1.019E−07
τ (ns)		92.50	54.84	55.05	55.57	58.57	72.34	101.9

Finally, the number of cycles (N) for photon absorption/emission should be maximized to decelerate the molecule sufficiently^43,44^. One can define N in terms of total decay channels involved (ɳ) as the following:

2 $${\text{N}} = \frac{1}{1 - \eta }$$

**Figure 5:**
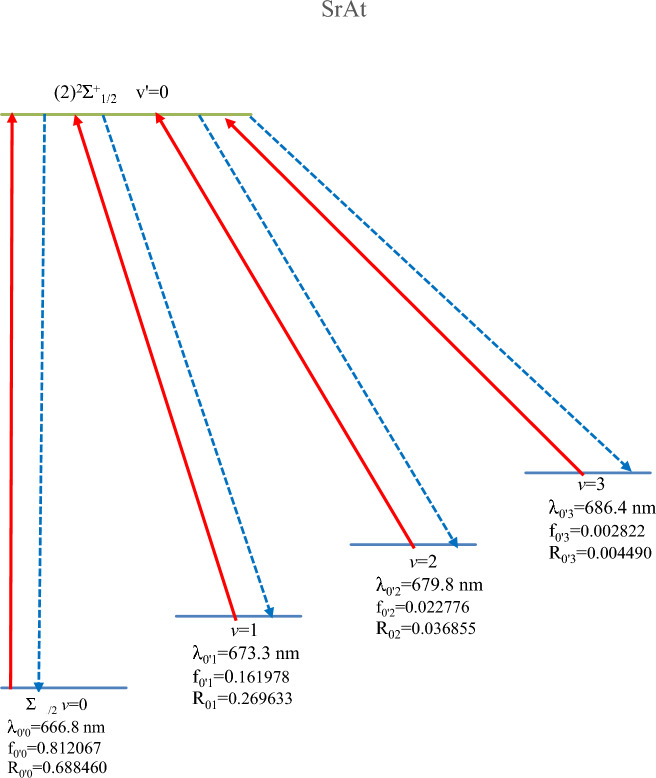
Laser cooling scheme with the transition X^2^$\sum_{1/2}^{ + }$ − (2)^2^$\sum_{1/2}^{ + }$ of the molecule SrAt.

In our case, we propose ɳ = R_0′0_ + R_0′1_ + R_0′2_ + R_0′3_, for which N = 1786. The corresponding laser cooling scheme is given in Fig. 5. The solid red lines represent the cycling lasers, while the dotted lines represent the spontaneous decay. The values of the vibrational transitions FCF (f_ν′ν_) and the vibrational branching ratios R_ν′ν_ are annotated under the ground state vibrational level involved in the corresponding transition. The proposed laser wavelengths are in the visible domain, with the primary pumping laser at λ_0′0_ = 666.8 nm, and the three repumping lasers used to close the leaks from higher vibrational levels at wavelengths λ_0′1_ = 673.3 nm, λ_0′2_ = 679.8 nm, λ_0′3_ = 686.4 nm.

The lowest SrAt temperature that can be reached through the Doppler and Sysphus laser cooling processes are in the order of the μK, as shown with the following corresponding experimental parameters needed below^43,45^:

3 $$V_{rms} = \frac{hN}{{m\lambda_{00} }} = {3}.{66}\;{\text{m}}/{\text{s}}$$

4 $${\text{T}}_{{{\text{ini}}}} = \frac{{mV^{2} }}{{2k_{B} }} = 0.{24}\;{\text{K}}$$

5 $${\text{a}}_{{{\text{max}}}} = \frac{{hN_{e} }}{{N_{tot} m\lambda_{00} \tau }} = {1}.{91} \times {1}0^{{3}} \;{\text{m}}/{\text{s}}^{{2}}$$

6 $$L_{min} = \frac{{k_{B} T_{ini} }}{{ma_{max} }}{3}.{\text{5 mm}}$$

7 $${\text{T}}_{{\text{D}}} = {\text{h}}/\left( {{4} \times \pi \times \tau \times {\text{k}}_{{\text{B}}} } \right) = {17}.{8}\;\mu {\text{K}}\;{\text{and}}\;{\text{T}}_{{\text{r}}} = {\text{h}}^{{2}} /\left( {{\text{m}} \times \lambda^{{2}}_{00} \times {\text{k}}_{{\text{B}}} } \right) = 0.{15}\;\mu {\text{K}}$$

where a_max_ and T_ini_ are the molecule’s maximum deceleration and initial temperature, respectively, and V_*rms*_ is the rms velocity. The parameters *m* and L_min_ are the molecule’s mass and minimum slowing distance, respectively. N_e_ is the number of excited states in the main cycling transition, and N_tot_ is the number of the excited states connected to the ground state plus N_e_. According to the SrAt laser cooling scheme, the ratio N_e_/N_tot_ equals 1/5, considering the vibrational ground and excited states. T_.D._ and T_r_ are, respectively, the Doppler and recoil temperatures. The slowing distance L_min_ is relatively small; however, a close scale (12 mm) stopping length has been proposed to slow down hydrogen atoms^44^.

Following the same investigation type, we considered the transition X^2^$\sum_{1/2}^{ + }$ − (1)^2^Π_1/2_ for the molecule BaAt. The FCF and the transition dipole moment for this transition are represented respectively in Figs. 3 and 4. This system shows a more evident FCF scheme diagonal feature compared to that of SrAt, where (v′,v) transitions among (0,0), (1,1) and (2,2) vibrational states have a higher probability compared to non-diagonal ones. The corresponding branching ratio values and radiative lifetime are given in Table 8. Several laser cooling loops can be built up for this molecule, with different numbers of cycles (N) for photon absorption/emission (Eq. 3). The number of cycles (N) and the corresponding schemes are given in Table 9, along with the corresponding experimental parameters (L, V_rms,_ a_max,_ and N_tot_). N_e_ was considered equal to one for all schemes.

**Table 8:** The vibrational Einstein Coefficients A_v′v_, the vibrational branching ratios R_v′v,_ and the radiative lifetimes τ for transitions between the electronic states (1)^2^Π_1/2_—X^2^$\sum_{1/2}^{ + }$ of the molecules BaAt.

BaAt								
		ν_′_((1)^2^Π_1/2_) = 0	1	2	3	4	5	6
ν ((X^2^$\sum_{1/2}^{ + }$)) = 0	A_νν′_	24,134,517.35	3,593,588.475	2.53E + 05	16,057.43464	1307.950081	9.560201603	6.16E + 01
	R_νν′_	0.88641120	0.13164116	0.00934283	0.00059800	0.00004898	0.00000038	0.00000389
ν = 1	A_νν′_	2,955,379.765	18,798,196.57	5.55E + 06	546,432.3912	43,238.75021	1769.024173	2.43E + 01
	R_νν′_	0.10854502	0.68861985	0.20484849	0.02034984	0.00161912	0.00007121	0.00000154
ν = 2	A_νν′_	132,424.2432	4,637,077.006	1.55E + 07	6,351,984.012	866,728.2549	68,486.78012	2.44E + 03
	R_νν′_	0.00486367	0.16986647	0.57095936	0.23655594	0.03245556	0.00275691	0.00015453
ν = 3	A_νν′_	4667.576649	256,465.6782	5.28E + 06	12,120,202.45	8,278,244.024	1,470,223.11	1.35E + 05
	R_νν′_	0.00017143	0.00939491	0.19483133	0.45137171	0.30998766	0.05918337	0.00855618
ν = 4	A_νν′_	232.6752906	11,922.48653	5.15E + 05	6,848,025.404	8,637,406.196	9,263,703.007	1.93E + 06
	R_νν′_	0.00000855	0.00043675	0.01901255	0.25502915	0.32343687	0.37290747	0.12187244
ν = 5	A_νν′_	2.687553238	1107.070168	2.53E + 04	907,480.6502	7,620,633.92	6,356,719.781	9.46E + 06
	R_νν′_	0.00000010	0.00004055	0.00093217	0.03379573	0.28536275	0.25588777	0.59809377
ν = 6	A_νν′_	0.874561527	8.501949302	1986.283649	61,749.86142	1,257,517.207	7,680,915.824	4,291,378.9
	R_νν′_	0.00000003	0.00000031	0.00007327	0.00229964	0.04708907	0.30919287	0.27131766
Sum(s^-1^) = A_ν′ν_		27,227,225.17	27,298,365.79	27,108,517.66	26,851,932.2	26,705,076.3	24,841,827.09	15,816,806.69
τ:(s) = 1/A_ν′ν_		3.67279E−08	3.66322E−08	3.68888E−08	3.72413E−08	3.74461E−08	4.02547E−08	6.32239E−08
τ:(ns)		36.73	36.63	36.89	37.24	37.45	40.25	63.22

**Table 9:** Variation of the laser slowing distance (L) in function of the number of the lasers needed (Laser N°), the number of cycles (N) for photon absorption/emission and the total decay channels involved (ɳ) for cooling BaAt and SrAt molecular beam.

BaAt molecule							
	Lasers N°	N	Total decay channels involved (ɳ)	L	V (m/s)	a_max_ (m/s^2^)	N_tot_
A	4	115 254	R_0′0_ + R_0′1_ + R_0′2_ + R_0′3_	1.36 m	128.42	6.07 × 10^3^	5
B	3	555.3	R_0′0_ + R_0′1_ + R_0′2_	2.52 mm	6.19	7.58 × 10^3^	4
C	4	90,242	(R_0′0_ + R_0′1_) + (R_0′2_ + R_0′3_)( R_1′0_ + R_1′1_ + R_1′2_ + R_1′3_	83.3 cm	100.55	6.07 × 10^3^	5
SrAt molecule							
		1786	R_0′0_ + R_0′1_ + R_0′2_ + R_0′3_	0.35 mm	3.66	1.91 × 10^3^	5

The values of a_max_ and N_tot_ are mentioned for each scheme.

**Figure 6:**
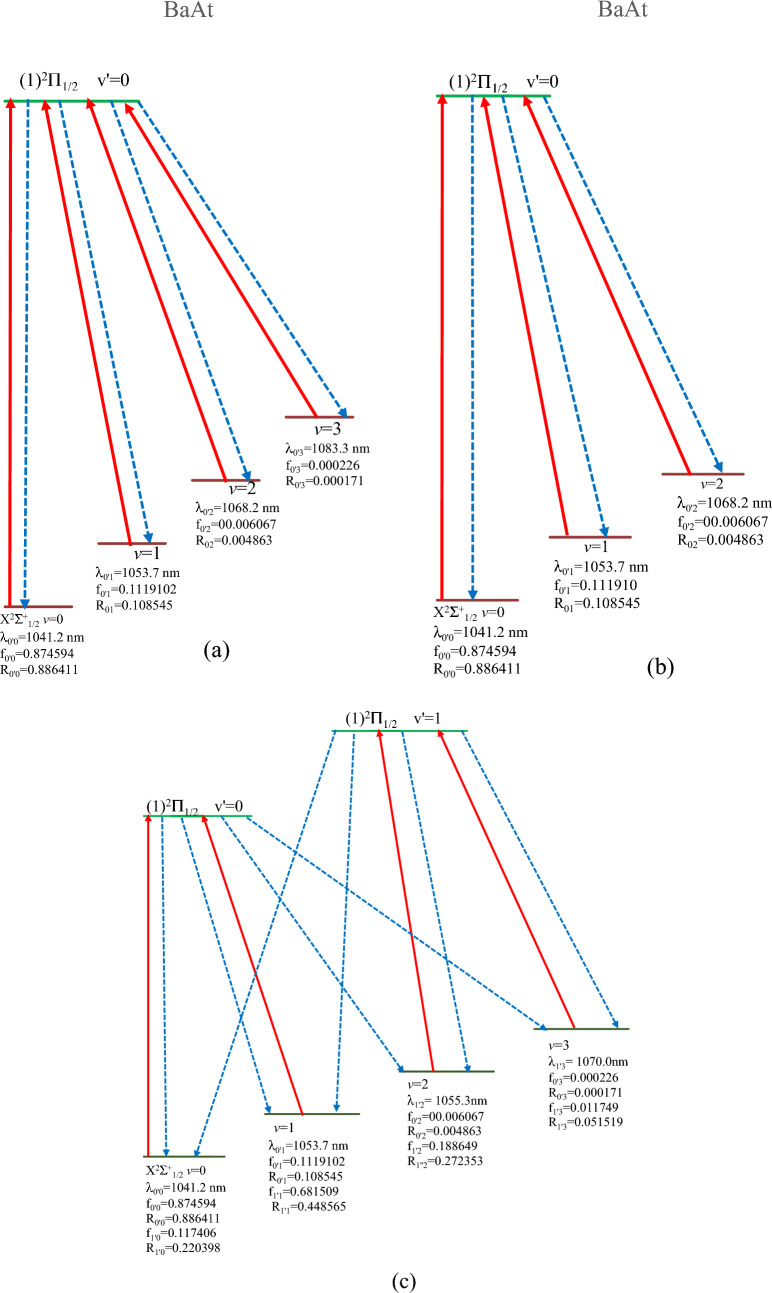
Laser cooling schemes with the transition X^2^$\sum_{1/2}^{ + }$ − (1)^2^Π_1/2_ of the molecule BaAt.

The slowing distances of the three schemes are within the experimental conditions for the cooling of a molecule, as they range between 2.52 mm and 1.36 m. The laser cooling scheme (A) is represented in Fig. 6a, where the solid red lines represent the driven lasers, and the dotted lines represent the spontaneous decays. Their main pumping and repumping laser wavelengths, in addition to the FCF (f_ν′ν_) and the vibrational branching ratios R_ν′ν_ among different transitions, are also represented. The wavelength of the primary pumping laser is λ_0′0_ = 1041.2 nm, and those of the repumping laser are λ_0′1_ = 1053.7 nm, λ_0′2_ = 1068.2 nm, and λ_0′3_ = 1083.3 nm in the near-infrared region. The graphical representation of the scheme (B) (by using three lasers) is given in Fig. 6b. Scheme (C) represents another suggested scheme with four lasers for the molecule BaAt, given in Fig. 6c. This last scheme presents new pumping lasers whose wavelengths are λ_1′2_ = 1055.3 nm and λ_1′3_ = 1070.0 nm. The lowest attainable Doppler and recoil temperatures for BaAt are T_D_ = 104.0 μK, and T_r_ = 51.9 nK among all three schemes as they only depend on the value of τ and λ_00_ for a given molecule.

## Conclusion

The MRCI + Q technique allowed the investigation of 63 electronic states with and without considering the spin–orbit coupling effect of the doublet and quartet electronic states of SrAt and BaAt molecules. The adiabatic potential energy curves and the static dipole moment curves have been plotted for these electronic states. The spectroscopic constants ${{\text{T}}}_{{\text{e}}}$, R_e_, ${\upomega }_{{\text{e}}}$, B_e_ were deduced here for the first time to the best of our knowledge. The results are compatible with our previously published work of molecules containing alkaline earth metals and halogens, obtained using the same calculation method^25,26^. Based on the canonical function approach, the values of the ro-vibrational constants E_v_, B_v_, D_v_, with the abscissas of turning points R_min_ and R_max,_ have been calculated for the ground and some low-lying excited states of the BaAt molecule. Transition parameters such as the FCFs, the radiative lifetime, the branching ratio, and the experimental parameters for the molecules SrAt and BaAt confirm their candidacy for Doppler and Sysphus laser cooling. The proposed laser cooling schemes may open the way for new laser cooling experiments.

### Supplementary Information

Supplementary Figures.

## Data Availability

The datasets used and/or analyzed during the current study are available from the corresponding author upon reasonable request.
