# Supplementary material for: Theoretical electronic structure with spin–orbit coupling effect of the molecules SrAt and BaAt for laser cooling studies
Source: Sci Rep. 2024 Mar 15;14:6289. doi: 10.1038/s41598-024-53564-5 (PMC10943126; doi:10.1038/s41598-024-53564-5)
Supplement: Supplementary file 1 — Supplementary Figures. [file 41598_2024_53564_MOESM1_ESM.doc]

**Supplementary Material**

- Figures (FS1-FS9): Potential energy curves for the lowest doublet and quartet electronic states of the five molecules BeAt, MgAt, CaAt, SrAt and BaAt without spin-orbit coupling
- Figures (FS10-FS15): Static dipole moment curves for the lowest doublet and quartet electronic states of the five molecules BeAt, MgAt, CaAt, SrAt and BaAt without spin-orbit coupling

FS1: The potential energy curves of the doublet states of BeAt molecule.

FS2: The potential energy curves of the quartet states of BeAt molecule.


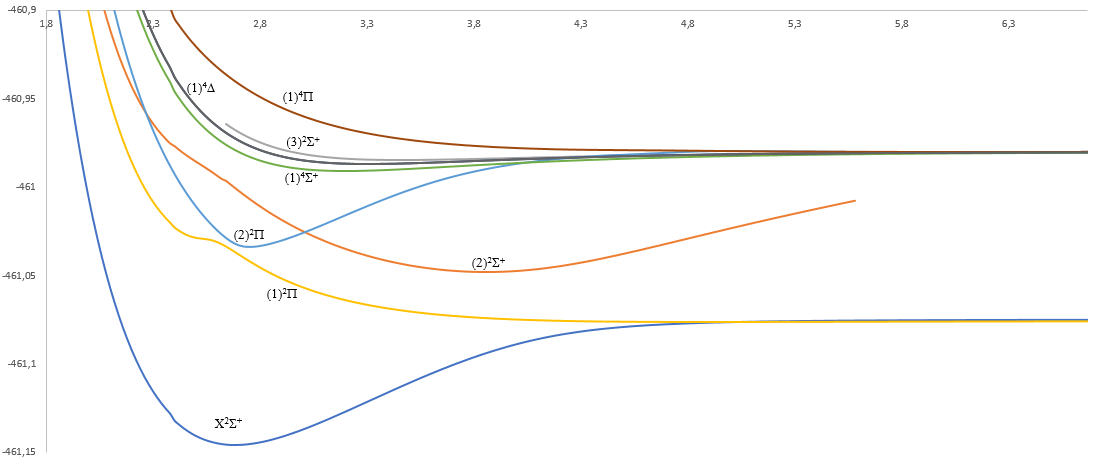


FS3: The potential energy curves of the doublet and quartet states of MgAt molecule.

FS4: The potential energy curves of the doublet and quartet states of CaAt molecule.

FS5: The potential energy curves of the doublet states of SrAt molecule.

FS6: The potential energy curves of the quartet states of SrAt molecule.

FS7: The potential energy curves of the doublet states of BaAt molecule.

FS8: The potential energy curves of the quartet states of BaAt molecule.

FS9: The permanent dipole moment curves of the doublet states of BeAt molecule.

FS10: The permanent dipole moment curves of the quartet states of BeAt molecule.

FS11: The permanent dipole moment curves of the doublet states of MgAt molecule.

FS12: The permanent dipole moment curves of the quartet states of MgAt molecule.

FS13: The permanent dipole moment curves of the doublet and quartet states of CaAt molecule.

FS14: The permanent dipole moment curves of the doublet and quartet states of SrAt molecule.

FS15: The permanent dipole moment curves of the doublet and quartet states of BaAt molecule.
